# Bibliographical Notices

**Published:** 1844-05

**Authors:** 


### BIBLIOGRAPHICAL NOTICES.

*Fifth Annual Report of the Directors and Superintendent of the Ohio Lunatic Asylum.*

This is an able document, containing a mass of statistical information of the highest interest to the Physician, the Legislator and the Philanthropist.

The tabular statements of the Superintendent—William M. Awl, M. D.,—of the causes of lunacy, duration and results of treatment, &c., are at once comprehensive and satisfactory.

We subjoin a summary of the operations of the Asylum, for the five years of its existence.

| | | | |
|---|---|---|---|
| Whole number of patients admitted in five years | | | 473 |
| Males | | 248 | |
| Females | | 225—473 | |
| Old cases | | 259 | |
| Recent do. *(less duration than one year)* | | 214—473 | |
| Paupers | | 349 | |
| Pay patients | | 124—473 | |
| Single | | 226 | |
| Married | | 203 | |
| Widows | | 33 | |
| Widowers | | 11—473 | |
| Whole number discharged | Recovered | 203 | |
| | Improved | 18 | |
| | Incurable | 51 | |
| | Idiotic | 2 | |
| | Died | 51—325 | |
| Whole number of recent cases discharged | Recovered | 154 | |
| | Incurable | 4 | |
| | Died | 17—175 | |
| Whole number of old cases discharged | Recovered | 49 | |
| | Incurable | 67 | |
| | Died | 34—150–325 | |

Per ct. of recoveries on all cases admitted in 5 years. . . . . . . .42.91
Per ct. of recoveries on all cases discharged in 5 years. . . . . .62.46
Per ct. of recoveries on all recent cases discharged in 5 years .88.00
Per ct. of recoveries on all old cases discharged in 5 years. . .32.66
Per ct. of deaths on the whole number, (51 of 473). . . . . . . .10.70
Average per ct. of deaths in 5 years. . . . . . . . . . . . . . . . . . . . 8.65

The following is also interesting, as showing the relative number of patients afflicted with different species of insanity.

Mania, 308; do. epileptic variety, 30; do. homicidal do. 5; Melancholia, 63; do. suicidal variety, 21; Moral insanity, 15; Dementia, 28; Idiotism or imbecility, 3.—Total, 473.

Notwithstanding the great number who have received the benefit of the institution, the demands upon it appear to be far beyond the means of accommodation. Two additional buildings are in progress of erection, for the accommodation of incurables; an act of the Legislature of Ohio, having appropriated $20,000 in cash, and $25,000 in convict labor, for that purpose.

From many general remarks replete with sound sense, and evincing a discriminating judgment and a tact of observation highly complimentary to their possessor, we cannot forbear quoting the following. After mentioning several apartments "to be built as strong and secure as massive limestone and mortar can make them," Dr. Awl remarks:

"These rooms are intended for furious maniacs, of extraordinary strength and malevolent dispositions; or, if the Legislature think proper, for the special reception and detention of dangerous homicides, where they can be safely and faithfully examined, by those who are familiar with mental disorders, when the courts have doubt in regard to the fact of insanity.

"The great interest which is at this time felt upon the subject of *homicidal insanity*, by the legal and medical professions, as well as the public at large, and the evident necessity of having some plan by which the important question of mental soundness and moral responsibility, in criminal cases, may be safely and certainly decided, have induced us to prepare the strong apartments aforementioned; and we respectfully propose this method for the consideration of the public authorities in our State.

"Unquestionably, the premeditating, cold-blooded murderer, deserves to forfeit his miserable life upon the gallows; but the prostration, impairment, or loss of heaven-born reason in a fellow being, as certainly removes moral accountability, and excuses crime. The only difficulty, for either the jurist or the physician, is to be able to determine the fact of mental derangement with clearness and satisfaction; and this, with becoming respect, we think can be decided with the greatest certainty, in an institution devoted to the subject, where the officers have all the advantages of experience, and the best means of acquiring information."

---

ADDRESS ON INSANITY AND THE ESTABLISHMENT OF A LUNATIC ASYLUM. Delivered Dec. 25, 1843, in the M. E. Church, before the Committee of the House of Representatives on Education, and the public. By JOHN EVANS, M. D., of Attica, Ia.,

The object of this address, as intimated in its caption, is to direct public opinion to the wants of the insane in the State of Indiana, and to call the attention of the Legislature to their proper relief.

The "Address" gives a rapid sketch of the history of the treatment of lunatics, and "a synopsis of the important points of the *moral treatment.*" It also makes strong appeals to the humanity of the citizens and the policy of the Legislative authorities. The imposibility of benefit to the insane under the existing laws of the State, is ably shown. Only pauper lunatics are provided for, and those only in a way without possibility of effective treatment.

A calculation from data, derived from authoritative sources, shows that $30,000 would be sufficient to build and furnish an Asylum for the accommodation of 100 patients. "A tax of one cent upon $100 valuation of property in the State, would raise the amout in three years." After this, Dr. E., remarks: "There is no question up whether we will take care of our lunatic paupers or not, nor whether a tax shall be laid for that purpose, for we cannot avoid either, as the counties must provide for them at the expense of the public, if the State does not. And how it is attended to by the counties we have already seen. The only question is, will we adhere to the present yet *miserable* plan, or will we adopt the one dictated by the strongest considerations of economy, and the purest principles of humanity."

We have quoted the above, as it is equally applicable to the condition of things in the State of Illinois. The only provision for the insane of this State, is in "An act regulating the estates of Idiots, Lunatics and persons distracted, and for other purposes," approved Feb. 12, 1823. In this act, (Sec. 6.) provision is made for constraint and maintainance, but none for *cure.* In Dr. Evans' address, we find quoted the authority of "Dr. Woodward, of the Massachusetts Lunatic Hospital," who "demonstrated that the average cost of maintaining 25 recent cases, both before they entered and during their residence in the Hospital, was $50 each, while that of supporting as many of the oldest cases was $1903 each." "This shows," (we quote from the address,) "a difference of more than thirty dollars to one in favor of curing cases, instead of keeping them on hand." It is to be hoped that a due consideration of these facts will cause medical gentlemen to urge, and the Legislative bodies in Illinois, to effect a reform in these respects, a reform called for by humanity and policy. We would be glad to see Dr. Evans' address widely distributed, and generally read.—ED.

---

*Review of a Pamphlet, entitled* "PHYSIOLOGY VINDICATED *in a Critique on Liebig's Animal Chemistry.* By CHARLES CALDWELL, M. D." By ROBERT PETER, M. D.

In this Review, the prejudices and antiquated views of the venerable Professor, who is the author of the "Critique," are viewed through the clear medium of modern science. The opening sentence of Dr. Peter, will explain more accurately than we could do, the design of the Review.

"The avowed design of the author of the pamphlet in hand, as given in his preface, is "*conservative* rather than *promotive*—to prevent the science of physiology, in whose behalf it was conceived and resolved on, from being *injured and degraded*, rather than actually to IMPROVE and ELEVATE it;" and the belief that its publication will *not* tend to improve and elevate knowledge in general, or any branch of science in particular, but that it will rather injure and degrade; with the conviction that it is my duty to oppose, to the extent of my abilities, all such tendencies, from whatever quarter, is the strong reason which urges me to offer the following remarks to the medical public."

The Review is spirited, and shows in the Professor, without any appearance of design, depth of research upon the subjects discussed, and a zeal for the advancement and elevation of the science which he defends.

The "Critique" of Dr. Caldwell has never come into our hands, but from the extracts given, it is evident that the reviewer has the stronger side of the question, nor has it lost ground in his hands. It must be confessed that Dr. P. handles his opponent "without gloves;" witness the following extract:

"In undertaking, in the present instance, the disagreeable task of exposing error, many motives are presented to induce me to prefer the ease of silence. The venerable age and acknowledged standing of the author; the untiring ability with which he wields his pen, and the most ready use of argument to sustain his positions, so as often to make the "worse *appear* the *better* reason;" the consideration that the work which he attacks in the present pamphlet, cannot be put down by a mere clash of logical arms, by the most ingenious mis-statement of its propositions, nor the strongest array of perverted or misquoted facts; the belief that any man of sense, or of clear unbiassed judgment, who had studied the productions of Liebig, would at once, without my assistance, perceive the injustice done to truth, logic, and that author, in the pamphlet of Professor Caldwell; and lastly, but not least, the fact, that in the performance of the task I have assumed, I shall be obliged to convict the Professor, not only of practical adherence to the old mode of philosophizing, namely, that of the school of Aristotle, but also of wilful or ignorant misconstruction of facts and arguments; and, what is more disagreeable, I shall be forced to expose, in his production, an amount of ignorance of science in general, and even of physiology, whose cause he undertakes to *vindicate*, and of which he has been professedly a teacher for so great a number of years, as would disgrace a tyro, and must appear incredible to the common observer."—Ed.

